# Supervised Regularized Canonical Correlation Analysis: integrating histologic and proteomic measurements for predicting biochemical recurrence following prostate surgery

**DOI:** 10.1186/1471-2105-12-483

**Published:** 2011-12-19

**Authors:** Abhishek Golugula, George Lee, Stephen R Master, Michael D Feldman, John E Tomaszewski, David W Speicher, Anant Madabhushi

**Affiliations:** 1 Department of Electrical and Computer Engineering, Rutgers University, Piscataway, New Jersey, USA; 2 Department of Biomedical Engineering, Rutgers University, Piscataway, New Jersey, USA; 3 Department of Pathology, University of Pennsylvania, Philadelphia, Pennsylvania, USA; 4 The Wistar Institute, Philadelphia, Pennsylvania, USA

## Abstract

**Background:**

Multimodal data, especially imaging and non-imaging data, is being routinely acquired in the context of disease diagnostics; however, computational challenges have limited the ability to quantitatively integrate imaging and non-imaging data channels with different dimensionalities and scales. To the best of our knowledge relatively few attempts have been made to quantitatively fuse such data to construct classifiers and none have attempted to quantitatively combine histology (imaging) and proteomic (non-imaging) measurements for making diagnostic and prognostic predictions. The objective of this work is to create a common subspace to simultaneously accommodate both the imaging and non-imaging data (and hence data corresponding to different scales and dimensionalities), called a metaspace. This metaspace can be used to build a meta-classifier that produces better classification results than a classifier that is based on a single modality alone. Canonical Correlation Analysis (CCA) and Regularized CCA (RCCA) are statistical techniques that extract correlations between two modes of data to construct a homogeneous, uniform representation of heterogeneous data channels. In this paper, we present a novel modification to CCA and RCCA, Supervised Regularized Canonical Correlation Analysis (SRCCA), that (1) enables the quantitative integration of data from multiple modalities using a feature selection scheme, (2) is regularized, and (3) is computationally cheap. We leverage this SRCCA framework towards the fusion of proteomic and histologic image signatures for identifying prostate cancer patients at the risk of 5 year biochemical recurrence following radical prostatectomy.

**Results:**

A cohort of 19 grade, stage matched prostate cancer patients, all of whom had radical prostatectomy, including 10 of whom had biochemical recurrence within 5 years of surgery and 9 of whom did not, were considered in this study. The aim was to construct a lower fused dimensional metaspace comprising both the histological and proteomic measurements obtained from the site of the dominant nodule on the surgical specimen. In conjunction with SRCCA, a random forest classifier was able to identify prostate cancer patients, who developed biochemical recurrence within 5 years, with a maximum classification accuracy of 93%.

**Conclusions:**

The classifier performance in the SRCCA space was found to be statistically significantly higher compared to the fused data representations obtained, not only from CCA and RCCA, but also two other statistical techniques called Principal Component Analysis and Partial Least Squares Regression. These results suggest that SRCCA is a computationally efficient and a highly accurate scheme for representing multimodal (histologic and proteomic) data in a metaspace and that it could be used to construct fused biomarkers for predicting disease recurrence and prognosis.

## Background

With the plentitude of multi-scale, multi-modal, disease pertinent data being routinely acquired for diseases such as breast and prostate cancer, there is an emerging need for powerful data fusion (DF) methods to integrate the multiple orthogonal data streams for the purpose of building diagnostic and prognostic meta-classifiers for disease characterization [1]. Combining data derived from multiple sources has the potential to significantly increase classification performance relative to performance trained on any one modality alone [2]. A major limitation in constructing integrated meta-classifiers that can leverage imaging (histology, MRI) and non-imaging (proteomics, genomics) data streams is having to deal with data representations spread across different scales and dimensionalities [3].

For instance, consider two different data streams *F_A_*(*x*) and *F_B_*(*x*) describing the same object *x*. If *F_A _*(*x*) and *F_B_*(*x*) correspond to the same scale or resolution and also have the same dimensionality, then one can envision, concatenating the two data vectors into a single unified vector [*F_A_*(*x*), *F_B_*(*x*)] which could then be used to train a classifier. However when *F_A_*(*x*) and *F_B_*(*x*) correspond to different scales, resolutions, and dimensionalities, it is not immediately obvious as to how one would go about combining the different types of measurements to build integrated classifiers to make predictions about the class label of *x*. For instance, directly aggregating data from very different sources without accounting for differences in the number of features and relative scaling, can not only lead to the *curse of dimensionality *(too many features and not enough corresponding samples [4]), but can lead to classifier bias towards the modality with more attributes. A possible solution is to first project the data streams into a space where the scale and dimensionality differences are removed; a *meta-space *allowing for a homogeneous, fused, multi-modal data representation.

DF methods try to overcome these obstacles by creating such a metaspace, on which a proper meta-classifier can be constructed. Methods leveraging embedding techniques have been proposed to try and fuse such heterogeneous data for the purpose of classification and prediction [2,3,5-7]. However, all of these DF techniques have their own weaknesses in creating an appropriate representation space that can simultaneously accommodate multiple imaging and non-imaging modalities. Generalized Embedding Concatenation [5] is a DF scheme that relies on dimensionality reduction (DR) methods to first eliminate the differences in scales and dimensionalities between the modalities before fusing them. However, these DR methods face the risk of extracting noisy features which degrade the metaspace [8]. Other variants of the embedding fusion idea, including Consensus embedding [6] and Boosted embedding [3] have yielded promising results, but come at a high computational cost. Consensus embedding attempts to combine multiple low dimensional data projections via a majority voting scheme while the Boosted embedding scheme leverages the Adaboost classifier [9] to combine multiple weak embeddings. In the case of weighted multi-kernel embedding using graph embedding [7] and support vector machine classifiers [2], insufficient training data can lead to overfitting and inaccurate weights to the various kernels, which can lower the performance of the meta-classifier [10].

CCA is a statistical DF technique that extracts linear correlations, by using cross-covariance matrices, between 2 data sources, *X *and *Y*. It capitalizes on the knowledge that the different modalities represent different sets of descriptors for characterizing the same object. For this reason, the mutual information that is most correlated between the two modalities will provide the most meaningful transformation into a metaspace. In recent years, CCA has been used to fuse heterogeneous data such as pixel values of images and the text attached between these images [11], assets and liabilities in banks [12], and audio and face images of speakers [13].

Regularized CCA (RCCA) is an improved version of CCA which in the presence of insufficient training data prevents overfitting by using a ridge regression optimization scheme [14]. Denote *p *and *q *as the number of features in *X *and *Y*, and *n *as the sample size. When *n *< <*p *or *n *< <*q*, the features in *X *and *Y *tend to be highly collinear. This leads to ill-conditioned matrices *C_xx _*and *C_yy_*, which denote the covariance matrix of *X *with itself and *Y *with itself, such that their inverses are no longer reliable resulting in an invalid computation of CCA and an unreliable metaspace [15]. The condition placed on the data to guarantee that *C_xx _*and *C_yy _*will be invertible is *n *≥ *p *+ *q *+ 1 [16]. However, that condition is usually not met in the bioinformatics domain, where samples (*n*) are usually limited, and modern technology has enabled very high dimensional data streams to be routinely acquired resulting in very high dimensional feature sets (*p *and *q*). This creates a need for regularization, which works by adding small positive quantities to the diagonals of *C_xx _*and *C_yy _*to guarantee their invertibility [17]. RCCA has been used to study expressions of genes measured in liver cells and compare them with concentrations of hepatic fatty acids in mice [18]. However, the regularization process required by RCCA is computationally very expensive. Both CCA and RCCA also fail to take complete advantage of class label information, when available [19].

In this paper, we present a novel efficient Supervised Regularized Canonical Correlation Analysis (SRCCA) DF algorithm that is able to incorporate a supervised feature selection scheme to perform regularization. Mainly, it makes better use of labeled information that in turn allows for significantly better stratification of the data in the metaspace. While SRCCA is more expensive than the overfitting-prone CCA, it provides the needed regularization while also being computationally cheaper than RCCA. SRCCA first produces an embedding of the most correlated data in both modalities via a low dimensional metaspace. This representation is then used in conjunction with a classifier (K-Nearest Neighbor [20] and Random Forest [21] are used in this study) to create a highly accurate meta-classifier.

Along with CCA and RCCA, SRCCA is compared with 2 other low dimensional data representation techniques: Principal Component Analysis (PCA) and Partial Least Squares Regression (PLSR). PCA [22] is a linear DR method that reduces high dimensional data to dominant orthogonal eigenvectors that try to represent the maximal amount of variance in the data. PLSR [23] is a DR method that uses one modality as a set of predictors to try to predict the other modality. Tiwari et al. [24] employed PCA in conjunction with a wavelet based representation of different MRI protocols to build a fused classifier to detect prostate cancer in vivo. PLSR has been used with heterogeneous multivariate signaling data collected from HT-29 human colon carcinoma cells stimulated to undergo programmed cell death to uncover aspects of biological cue-signal-response systems [25].

In this work, we apply SRCCA to the problem of predicting biochemical recurrence in prostate cancer (CaP) patients, following radical prostatectomy, by fusing histologic imaging and proteomic signatures. Biochemical recurrence is commonly defined as a detectable elevation of Prostate Specific Antigen (PSA), a key biomarker for CaP [26-28]. However, the nonspecificity of PSA leads to over-treatment of CaP, resulting in many unnecessary treatments, which are both stressful and costly [29-33]. Even the most widely used prognostic markers such as pathologist assigned Gleason grade [34], which attempts to capture the morphometric and architectural appearance of CaP on histopathology, has been found to be a less than perfect predictor of biochemical recurrence [35]. Additionally, Gleason grade has been found to be subject to inter-, and intra-observer variability [36-38]. While some researchers have proposed quantitative, computerized image analysis approaches [1,39,40] for modeling and predicting Gleason grade (a number that goes from 1 to 5 based on morphologic appearance of CaP on histopathology), it is still not clear that an accurate, reproducible grade predictor from histology will also be accurate in predicting biochemical recurrence and long term patient outcome [41].

Recent studies have shown that proteomic markers can be used to predict aggressive CaP [42,43]. Techniques such as mass spectrometry hold promise in their ability to identify protein expression profiles that might be able to distinguish more aggressive from less aggressive CaP and identify candidates for biochemical recurrence [44-46]. However, more and more, it is becoming apparent that a single prognostic marker may not possess sufficient discriminability to predict patient outcome which suggests that the solution might lie in an integrated fusion of multiple markers [47]. This then begs the question as to what approaches need to be leveraged to quantitatively fuse imaging and non-imaging measurements to build an integrated prognostic marker for CaP recurrence. The overarching goal of this study is to leverage SRCCA to construct a fused quantitative histologic, proteomic marker, and a subsequent meta-classifier, for predicting 5 year biochemical recurrence in CaP patients following surgery.

Our main contributions in this paper are:

• A novel data fusion algorithm, SRCCA, that builds an accurate metaspace representation that can simultaneously represent and accommodate two heterogeneous imaging and non-imaging modalities.

• Leveraging SRCCA to build a meta-classifier to predict risk of 5 year biochemical recurrence in prostate cancer patients following radical prostatectomy by integrating histological image and proteomic features.

The organization of the rest of the paper is as follows: In the methods section, we first review the 4 statistical methods, PCA, PLSR, CCA and RCCA. Next, we introduce our novel algorithm, Supervised Regularized Canonical Correlation Analysis (SRCCA). We then discuss the DF algorithm for metaspace creation and the computational complexities for CCA, RCCA and SRCCA. In the Experimental Design section, we briefly discuss the prostate cancer dataset considered in this study and the subsequent proteomic and histologic feature extraction schemes before moving on to the experiments performed on the dataset where we try to determine the ability of PCA, PLSR, CCA, RCCA and SRCCA to identify patients at risk for biochemical recurrence following surgery. The results are discussed in the subsequent section and the concluding remarks are presented at the end of the paper.

## Methods

### Review of PCA and PLSR

Principal Component Analysis (PCA) and Partial Least Squares Regression (PLSR) are common statistical methods used to analyze multi-modal data and they are briefly discussed in the following sections. However, further information, explaining how these two methods can be viewed as special cases of the generalized eigenproblem, can be found in [48].

#### Principal Component Analysis (PCA)

PCA [22] constructs a low dimensional subspace of the data by finding a series of linear orthogonal bases called principal components. Each component seeks to explain the maximal amount of variance in the dataset. Denote two multidimensional variables, *X *∈ ℝ^*n *× *p *^and *Y *∈ ℝ^*n*×*q*^, where *p *and *q *are the number of features in *X *and *Y *and *n *the number of overall samples. PCA is usually performed on the data matrix, *Z *∈ ℝ^*n*×(*p*+*q*)^, obtained by concatenating the individual modalities such that: *Z *= [*X Y*] [24]. $\bar{Z}\in {\mathbb{R}}^{n\;\times \;\left(p+q\right)}$ is then obtained by subtracting the means of all features for a certain sample from its original feature value in *Z *so that the resultant $\bar{Z}$ has rows with a 0 mean. $\bar{Z}$ is further broken using singular value decomposition into [22]:

(1) $$\bar{Z}=UEV^{T}$$

where *E *∈ ℝ^*n*×*n *^is a diagonal matrix containing the eigenvalues of the eigenvectors which are stored in *U *∈ ℝ^*p*×*p*^, and *V*^*T *^∈ ℝ^*m*×*n*^. The eigenvalues stored in *E *explain how much variance of the original $\bar{Z}$ is stored in the corresponding eigenvector, or principal component. Using these eigenvalues as a rank, the top d embedding components can be chosen to best represent the original data in a lower dimensional subspace.

#### Partial Least Squares Regression(PLSR)

PLSR [49] is a statistical technique that generalizes PCA and multiple regression. The general underlying model behind PLSR is [23]:

(2) $$X=TP^{T}+E$$

(3) $$Y=TC^{T}+F$$

where *T *∈ ℝ^*n*×*l *^is a score matrix, *P *∈ ℝ^*p*×*l *^and *C *∈ ℝ^*q*×*l *^are loading matrices for *X *and *Y*, and *E *∈ ℝ^*n*×*p *^and *F *∈ ℝ^*n*×*p *^are the error terms. PLSR is an iterative process and works by continually approximating, and improving the approximation of the matrices *T*, *P *and *C *[50].

### Review of CCA and RCCA

#### Canonical Correlation Analysis (CCA)

CCA [51] is a way of using cross-covariance matrices to obtain a linear relationship between the two multidimensional variables, *X *∈ ℝ^*n*×*p *^and *Y *∈ ℝ^*n*×*q*^. CCA obtains two directional vectors *w_x _*∈ ℝ^*p*×1 ^and *w_y _*∈ ℝ^*q*×1 ^such that *Xw_x _*and *Yw_y _*will be maximally correlated. It is defined as the optimization problem [11]:

(4) $$\rho =\underset{w_{x},w_{y}}{\max}\frac{w_{x}^{T}C_{xy}w_{y}}{\sqrt{w_{x}^{T}C_{xx}w_{x}w_{y}^{T}C_{yy}w_{y}}}$$

where *C_xy _*∈ ℝ^*p*×*q *^is the covariance matrix of the matrices *X *and *Y*, *C_xx _*∈ ℝ^*p*×*p *^is the covariance matrix of the matrix *X *with itself and *C_yy _*∈ ℝ^*q *× *q *^is the covariance matrix of the matrix *Y *with itself. The solution to CCA reduces to the solution of the following two generalized eigenvalue problems [52]:

(5) $$C_{xy}C_{yy}^{-1}C_{yx}=\lambda C_{xx}w_{x}$$

(6) $$C_{yx}C_{xx}^{-1}C_{xy}=\lambda C_{yy}w_{y}$$

where *λ *is the generalized eigenvalue representing the canonical correlation, and *w_x _*and *w_y _*are the corresponding generalized eigenvectors. CCA can further produce exactly min{*p*, *q*) orthogonal embedding components (sets of *w_x_X *and *w_y_Y*) which can be sorted in order of decreasing correlation, *λ*.

#### Regularized Canonical Correlation Analysis (RCCA)

RCCA [53,54] corrects for noise in *X *and *Y *by first assuming that *X *and *Y *are contaminated with noise, *N_x _*∈ ℝ^*n*×*p *^and *N_Y _*∈ ℝ^*n*×*q*^. We assume that these noise vectors in the *p *and *q *columns of *N_X _*and *N_Y_*, respectively, are gaussian, independent and identically distributed. For this reason, all combinations of the covariances of the *p *columns of *N_X _*and *q *columns of *N_Y _*will be 0 except the covariance of a particular column vector with itself. This variance of each column of *N_X _*and *N_Y _*is labeled *λ_x _*and *λ_y _*and these labels are called the regularization parameters. The matrix *C_xy _*will not be affected but the matrices *C_xx _*and *C_yy _*become *C_xx _*+ *λ_x _I_x _*and *C_yy _*+ *λ_x _I_x_*. The solution to RCCA now becomes the solution to these generalized eigenvalue problems [52]:

(7) $$C_{xy}{\left(C_{yy}+\lambda_{y}\;I_{y}\right)}^{-1}C_{yx}=\lambda \left(C_{xx}+\lambda_{x}\;I_{x}\right)w_{x}$$

(8) $$C_{yx}{\left(C_{xx}+\lambda_{x}\;I_{x}\right)}^{-1}C_{xy}=\lambda \left(C_{yy}+\lambda_{y}\;I_{y}\right)w_{y}$$

The regularization parameters next have to be chosen. For *i *∈ {1, 2, . . . , *n*}, let $w_{x}^{i}$ and $w_{y}^{i}$ denote the weights calculated from RCCA when samples *X_i _*and *Y_i _*are removed. *λ_x _*and *λ_y _*are varied in a certain range *θ*_1 _≤ *λ_x_*, *λ_y _*≤ *θ*_2 _and chosen via a grid search [55] optimization of the following cost function [18]:

(9) $$\underset{\lambda_{x},\lambda_{y}}{\max}\;\left[corr\;\left({\left\{X_{i}w_{x}^{i}\right\}}_{i=1}^{n},\;{\left\{Y_{i}w_{y}^{i}\right\}}_{i=1}^{n}\right)\right]$$

where *corr *(·, ·) refers to the Pearson's correlation coefficient [56]. The above cost function essentially measures the change in the produced $w_{x}^{i}$ and $w_{y}^{i}$ when a sample *i *is omitted and seeks the optimal *λ_x _*and *λ_y _*where this change is minimized. *λ_x _*and *λ_y _*are chosen using the embedding component with the highest *λ *and then adjusted for the remaining dimensions [18].

### Extending RCCA to SRCCA

Supervised Regularized Canonical Correlation Analysis (SRCCA) chooses *λ_x _*and *λ_y _*using a supervised feature selection method (*t*-test, Wilcoxon Rank Sum Test and Wilks Lambda Test are used in this study). Denote $W_{\text{1}}$ and $W_{2}$ as class 1 and class 2 and *μ*_1 _and *μ*_2_, $\sigma_{1}^{2}$ and $\sigma_{2}^{2}$, *n*_1 _and *n*_2 _as the means, variances, and sample sizes of $W_{\text{1}}$ and $W_{2}$. The data in the metaspace, *Xw_x _*or *Yw_y_*, can be split using its labels into the *n*_1 _samples that belong to $W_{\text{1}}$ and the *n*_2 _samples that belong to class $W_{2}$, where *n*_1 _+ *n*_2 _= *n*. These two partitions can then be used to calculate the discrimination level between the samples of the two classes in the metaspace representation. In this study, we implement RCCA with the *t*-test (SRCCA*_TT_*), the Wilcoxon Rank Sum Test (SRCCA*_WRST_*) and the Wilks Lambda Test (SRCCA*_WLT_*) to try to choose more appropriate regularization parameters, *λ_x _*and *λ_y_*, that can more successfully stratify the samples in the metaspace compared to the parameters chosen by RCCA. Similar to RCCA, for SRCCA, *λ_x _*and *λ_y _*are chosen using the embedding component with the most discriminatory score as chosen by the feature selection schemes below and then adjusted for the remaining dimensions.

#### SRCCA_TT_

The *t*-test [57] is a parametric test that assumes the distributions of the two samples are normal and tests whether these distributions have the same means. The *t*-score, which measures the number of standard deviations the two means of *n*_1 _samples of $W_{\text{1}}$ and *n*_2 _samples of $W_{2}$ are away from each other, is maximized using a grid search algorithm as:

(10) $$\underset{\lambda_{x},\lambda_{y}}{\max}\frac{\left||\mu_{1}-\mu_{2}|\right|}{\sqrt{\frac{\sigma_{1}^{2}}{n_{1}}+\frac{\sigma_{2}^{2}}{n_{2}}}}.$$

#### SRCCA_WRST_

Wilcoxon Rank Sum Test [58] sorts both the samples in order from lowest value to highest value. It then uses their respective ranks within the population to calculate the discriminatory score:

(11) $$\underset{\lambda_{x},\lambda_{y}}{\max}\left\{\left(\overset{n_{2}}{\underset{i=1}{\sum }}b_{i}-\frac{n_{2}\left(n_{2}+1\right)}{2}\right)\;,\;\left(n_{1}n_{2}-\overset{n_{2}}{\underset{i=1}{\sum }}b_{i}+\frac{n_{2}\left(n_{2}+1\right)}{2}\right)\right\},$$

where *b_i _*represents the rank of the sample $i\;\in \;W_{2}$ with respect to the rest of the samples.

#### SRCCA_W LT_

In an ideal metaspace representation, samples from each class will be grouped together while the samples from different classes will be grouped separately. The WLT [59] capitalizes on this knowledge and calculates the ratio of within class variance of both samples to the total variance of both samples combined. Wilks Lambda (Λ) is minimized using a grid search algorithm as:

(12) $$\underset{\lambda_{x},\lambda_{y}}{\min}\frac{n_{1}\sigma_{1}^{2}+n_{2}\sigma_{2}^{2}}{n\sigma^{2}}.$$

### Data Fusion in the context of CCA, RCCA and SRCCA

DF is performed as described in Foster et al. [60]. When the *Xw_x _*and *Yw_y _*are maximally correlated, each modality represents similar information, and thus either *Xw_x _*or *Yw_y _*can be used to represent the original two modalities in the metaspace. Moreover, *X *and *Y *are both descriptors of the same object and thus, the most relevant information is the data that exists and is correlated in both modalities. Thus, a high correlation of *Xw_x _*and *Yw_y _*is indicative that meaningful data, measuring the object of interest, is being added to the metaspace.

In order of decreasing *λ*, the top d embedding components, up to *φ *= min{*p*, *q*} can be chosen to represent the two modalities in a metaspace. However, the lower embedding components will have a lower *λ*, and thus a lower correlation between *Xw_x _*and *Yw_y _*which might imply that non-relevant data is being added to the metaspace. To avoid this issue, a threshold, *λ*_0_, can be selected such that only embedding components with *λ *≥ *λ*_0 _will be included in the metaspace.

### Computational Complexity

Given *φ *= min{*p*, *q*}, CCA has a computational complexity of *φ*! (based on the source code in [61]). The regularization algorithm requires a grid search process for each ordered pair (*λ_x_*, *λ_y_*). Assume *v *potential *λ_x _*and *λ_y _*sampled evenly between *θ*_1 _and *θ*_2_. RCCA requires a training/testing cross-validation strategy, at each ordered pair (*λ_x_*, *λ_y_*), to find the optimal *λ_x _*and *λ_y_*. It will require CCA to be performed an order of *n *times at each of the *v *intervals leading to a complexity of *vnφ*!. SRCCA only requires a CCA factorization once at each of the *v *intervals leading to a complexity of *vφ*!.

The computational complexities for each of the CCA schemes are summarized in Table 1. Table 1 indicates that SRCCA is an order of *n *times faster compared to RCCA. However, SRCCA is also more complex compared to CCA and will have a longer execution time.

**Table 1:** The computational complexities of all 3 DF methods used in this study

Method	Complexity
CCA	*φ*!

RCCA	*vnφ*!

SRCCA	*vφ*!

*φ *= min{*p*, *q*}, which represents the number of features in the lower dimensional modality, *n *is the sample size and *v *is the interval spacing over which *λ*_1 _and *λ*_2 _will be chosen in the range {*θ*_1_, *θ*_2_}.

### Experimental Design

#### Data Description

A total of 19 prostate cancer patients at the Hospital at the University of Pennsylvania were considered for this study. All patient identifiers are stripped from the data at the time of acquisition. The data was deemed to be exempt for review by the internal review board at Rutgers University and the protocol was approved by the University of Pennsylvania internal review board. Hence, the data was deemed eligible for use in this study. All of these patients had been found to have prostate cancer on needle core biopsy and subsequently underwent radical prostatectomy. 10 of these patients had biochemical recurrence within 5 years following surgery (BR) and the other 9 did not (NO BR). The 19 patient studies were randomly chosen from a larger cohort of 110 patient studies at the University of Pennsylvania all of whom had been stage and grade matched (Gleason score of 6 or 7) and had undergone gland resection. Of these 110 cases, 55 had experienced biochemical recurrence within 5 years while the other 55 had not. The cost of the mass spectrometry to acquire the proteomic data limited this study to only 19 patient samples. Following gland resection, the gland was sectioned into a series of histological slices with a meat cutter. For each of the 19 patient studies, a representative histology section on which the dominant tumor nodule was observable was identified. Mass Spectrometry was performed at this site to yield a protein expression vector. The representative histologic sections were then digitized at 40 × magnification using a whole slide digital scanner.

In the next two sections, we briefly describe the construction of the proteomic and histologic feature spaces. Subsequently we describe the strategy for combination of quantitative image descriptors from the tumor site on the histological prostatectomy specimen and the corresponding proteomic measurements obtained from the same tumor site, via mass spectrometry. The resultant meta-classifier, constructed in the fused meta-space, is then used to distinguish the patients at 5 year risk of biochemical recurrence following radical prostatectomy from those who are not.

#### Proteomic Feature Selection

Prostate slides were deparaffinized, and rehydrated essentially as described in [62]. Tumor areas previously defined on a serial H&E section were collected by needle dissection, and formalin cross-links were removed by heating at 99°C. The FASP (Filter-Aided Sample Preparation) method [63] was then used for buffer exchange and tryptic digest. After peptide purification on C-18 StageTips [64] samples were analyzed using nanoflow C-18 reverse phase liquid chromatography/tandem mass spectrometry (nLC-MS/MS) on an LTQ Orbitrap mass spectrometer. A top-5 data-dependent methodology was used for MS/MS acquisition, and data files were processed using the Rosetta Elucidator proteomics package, which is a label-free quantitation package that uses extracted ion chromatograms to calculate protein abundance rather than peptide counts. A high dimensional feature vector was obtained, denoted *ϕ*^*P *^∈ ℝ^19 × 953^, characterizing each patient's protein expression profile following surgery. This data underwent quantile normalization, log(2) transformation, and mean and variance normalization on a per-protein basis.

#### Quantitative Histologic Feature Extraction

**Figure 1:**
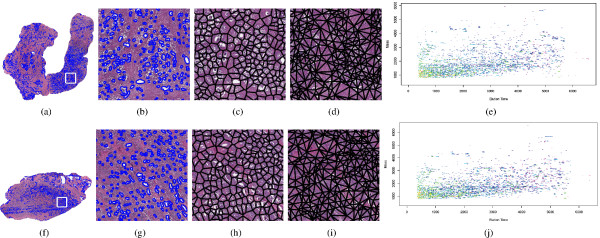
**Multi-modal patient data (top row: relapsed case, bottom row: non-relapsed case)**. (a), (f) Original prostate histology section showing region of interest, (b), (g) Magnified ROI showing gland segmentation boundaries, (c), (h) Voronoi Diagram (d), (i) Delaunay Triangulation depicting gland architecture, (e), (j) Plot of the proteomic profile obtained from the dominant tumor nodule regions (white box in (a), (f) respectively) via mass spectrometry.

In prostate whole-mount histology, denoted *ϕ*^*H *^∈ ℝ^19 × 151 ^(Figure 1 (a), (f)), the objects of interest are the glands (shown in Figure 1 (b), (g)), whose shape and arrangement are highly correlated with cancer progression [1,39,65,66]. We briefly describe this process below. Prior to extracting image features, we employ an automatic region-growing gland segmentation algorithm presented by Monaco et al. [67]. The boundaries of the interior gland lumen and the centroids of each gland, allow for extraction of 1) morphological and 2) architectural features from histology as described briefly below. More extensive details on these methods are in our other publications [5,39,68].

##### Glandular Morphology

The set of 100 morphological features [1], (denoted *ϕ*^*M *^∈ ℝ^19 × 100^), of attributes, consists of the average, median, standard deviation, and min/max ratio for features such as gland area, maximum area, area ratio, and estimated boundary length (See Table 2).

**Table 2:** Description of 25 Proteomic Features, 100 Morphological, and 51 Architectural

Proteomic	#	Description
Proteins Identified	25	Some include: CSNK2A1 protein, Dihydroxyacetone kinase,
		Dynamin-2, Glycogenin-1, Mitochondrial PDHA1, Mu-crystallin
		homolog, Nit protein 2, Nucleolin, Synaptonemal complex protein 1
		Putative uncharacterized protein RPL3

**Morphological**		**Description**

Gland Morphology	100	Area Ratio, distance Ratio, Standard Deviation of Distance,
		Variance of Distance, Distance Ratio, Perimeter, Ratio,
		Smoothness, Invariant Moment 1-7, Fractal Dimension, Fourier
		Descriptor 1-10 (Mean, Std. Dev, Median, Min/Max of each)

**Architectural**		**Description**

Voronoi Diagram	12	Polygon area, perimeter, chord length: mean, std. dev., min/max ratio, disorder

Delaunay Triangulation	8	Triangle side length, area: mean, std. dev., min/max ratio, disorder

Minimum Spanning Tree	4	Edge length: mean, std. dev., min/max ratio, disorder

Nearest Neighbors	27	Density of nuclei, distance to nearest nuclei

##### Architectural Feature Extraction

51 architectural image features, which have been shown to be predictors of cancer [69], (denoted *ϕ*^*A *^∈ ℝ^19 × 51^), were extracted in order to quantify the arrangement of glands present in the section (See Table 2). Voronoi diagrams, Delaunay Triangulation and Minimum Spanning Trees were constructed on the digital histologic image using the gland centroids as vertices, the gland centroids having previously been identified via the scheme in [68].

### Fusing Proteomic, Histologic Features for Predicting Biochemical Recurrence in CaP Patients Post-Surgery

#### Experiment 1 - Comparing SRCCA with CCA and RCCA

We performed CCA, RCCA, and SRCCA on selected multimodal combinations, *ϕ*^*P *^and *ϕ*^*J *^, where *J *∈ {*M*, *A*, *H*}. *ϕ*^*P *^was reduced to 25 features as ranked by the *t*-test, with a *p*-value cutoff of *p *= .05, using a leave-one-out validation strategy. For CCA, *ϕ*^*P *^and *ϕ*^*J *^were used as the two multidimensional variables, *X *and *Y*, as mentioned above in Section 2. For RCCA and SRCCA, *ϕ*^*P *^and *ϕ*^*J *^were used in a manner similar to CCA except they are tested with regularization parameters *λ_x _*and *λ_y _*evenly spaced from *θ*_1 _= .001 to *θ*_2 _= .2 with *v *= 200.

The top d = 3 embedding components (which were experimentally found to meet the criteria of *λ*_0 _= .99 for all SRCCA on all 3 multimodal combinations) were produced from CCA, RCCA, SRCCA*_TT_*, SRCCA*_WRST_*, and SRCCA*_WLT_*. The classification accuracies were determined with the classifiers K-Nearest Neighbor, denoted via *ϕ*^*KNN *^[20], with K = 1, and Random Forest, denoted via *ϕ*^*RF *^[21], with 50 Trees. Both these classifiers were used because of their high computational speed. Accuracies were determined using leave-one-out validation, which was implemented because of the small sample size. In this process, 18 samples were used for the initial feature pruning, determining the optimal regularization parameter and training the classifier while the remaining sample was used as the testing set for evaluating the classifier. This procedure was repeated till all the samples were used in the testing set.

#### Experiment 2 - Comparing SRCCA with PCA and PLSR

In addition to the steps performed in Experiment 1, metaspaces were also produced with PCA and PLSR. *ϕ*^*P *^and *ϕ*^*J *^were concatenated and PCA was then performed on this new data matrix. For PLSR, a regression of *ϕ*^*J *^on *ϕ*^*P *^was performed.

Similarly, using the top d = 3 embedding components produced from PCA, PLSR, SRCCA*_TT_*, SRCCA*_WRST_*, and SRCCA*_WLT_*, the classification accuracies of *ϕ*^*KNN *^, with K = 1, and *ϕ*^*RF *^, with 50 Trees, were determined using leave-one-out validation.

#### Experiment 3 - Comparing classifier accuracy for PCA, PLSR and CCA variants using metaspace representations

Using the 10 different values for d ∈ {1, 2, ..10}, and the 3 fusion schemes considered (*ϕ*^*P *^, *ϕ*^*M *^), (*ϕ*^*P *^, *ϕ*^*A *^), and (*ϕ*^*P *^, *ϕ*^*H *^), 30 different embeddings were obtained for PCA, PLSR, CCA, RCCA, SRCCA*_TT_*, SRCCA*_WRST_*, and SRCCA*_WLT_*. The maximum and median of these 30 different measurements for each classifier were calculated.

In addition, we denote as *α*_1_(*i*), the classification accuracy obtained by the DF scheme *i*, where *i *∈ {PCA, PLSR, CCA, RCCA} and *α*_2_(*j*) as the accuracy obtained by the DF scheme *i*, where *j *∈ {SRCCA*_TT_*, SRCCA*_WRST_*, SRCCA*_WLT_*}. A two paired student *t*-test was employed to identify whether there were statistically significant improvements in the 3 SRCCA variants by comparing the classification accuracies with the null hypothesis:

(13) $$H_{o}:\alpha_{1}\left(i\right)=\alpha_{2}\left(j\right)$$

for all *i *∈ {PCA, PLSR, CCA, RCCA} and for all *j *∈ {SRCCA*_TT_*, SRCCA*_WRST_*, SRCCA*_WLT_*}.

#### Experiment 4 - Computational consideration for RCCA and SRCCA

We measured the 3 individual single run completion times for RCCA and SRCCA to fuse (*ϕ*^*P *^, *ϕ*^*M *^), (*ϕ*^*P *^, *ϕ*^*A *^), and (*ϕ*^*P *^, *ϕ*^*H *^), with the null hypothesis:

(14) $$H_{o}:\text{completion}\;\text{time}\;\text{of}\;\text{RCCA}\;\text{ = }\;\text{completion}\;\text{time}\;\text{of}\;\text{SRCCA}$$

These experiments were performed on a quadcore computer with a clock speed of 1.8 GHz, and the programs were written on MATLAB(R) platform.

## Results and Discussion

### Experiment 1

Across both classifiers for d = 3, the 3 SRCCA variants, SRCCA*_TT_*, SRCCA*_WRST_*, SRCCA*_WLT_*, had a combined median classification accuracy of 80% compared to 60% for CCA and 42% for RCCA. SRCCA also performed better in all 36 of 36 direct comparisons with CCA and RCCA (see Tables 3 and 4). The higher classification accuracy results indicate that SRCCA produces a metaspace, where the samples are more stratified, compared to CCA and RCCA. This also seems to indicate that the supervised scheme of choosing regularization parameters, by the 3 SRCCA variants, is a more appropriate scheme for classification purposes compared to the ridge regression scheme used by RCCA.

**Table 3:** Experiment 1: Classification Accuracy with K-Nearest Neighbor

Dataset (*ϕ*^*P *^, *ϕ*^*J *^)	CCA	RCCA	SRCCA*_TT_*	SRCCA*_WRST_*	SRCCA*_WLT_*
(*ϕ*^*P *^, *ϕ*^*M *^)	53%	37%	**80**%	79%	79%

(*ϕ*^*P *^, *ϕ*^*A *^)	58%	47%	**74**%	68%	**74**%

(*ϕ*^*P *^, *ϕ*^*H *^)	63%	47%	**74**%	**74**%	**74**%

Classification accuracies obtained for fusing (*ϕ*^*P *^, *ϕ*^*M *^), (*ϕ*^*P *^, *ϕ*^*A *^), and (*ϕ*^*P *^, *ϕ*^*H *^), with CCA, RCCA, SRCCA*_TT_*, SRCCA*_WRST_*, and SRCCA*_WLT _*using the top d = 3 components, using *ϕ*^*KNN *^with K = 1 neighbor and leave-one-out validation to identify patients at the risk of biochemical recurrence from those who are not.

**Table 4:** Experiment 1: Classification Accuracy with Random Forest

Dataset (*ϕ*^*P *^, *ϕ*^*J *^)	CCA	RCCA	SRCCA*_TT_*	SRCCA*_WRST_*	SRCCA*_WLT_*
(*ϕ*^*P *^, *ϕ*^*M *^)	37%	42%	83%	81%	**84**%

(*ϕ*^*P *^, *ϕ*^*A *^)	74%	30%	81%	77%	**83**%

(*ϕ*^*P *^, *ϕ*^*H *^)	62%	42%	91%	89%	**93**%

Classification accuracies obtained for fusing (*ϕ*^*P *^, *ϕ*^*M *^), (*ϕ*^*P *^, *ϕ*^*A *^), and (*ϕ*^*P *^, *ϕ*^*H *^), with CCA, RCCA, SRCCA*_TT_*, SRCCA*_WRST_*, and SRCCA*_WLT _*using the top d = 3 components, using *ϕ*^*RF *^with 50 trees and leave-one-out validation to identify patients at the risk of biochemical recurrence from those who are not.

**Figure 2:**
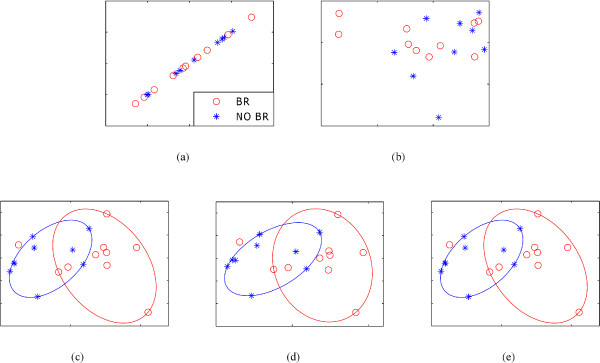
**2-dimensional representation of (*ϕ*^*P *^, *ϕ*^*A *^)**. 2-dimensional representation of (*ϕ*^*P *^, *ϕ*^*A *^) using (a) CCA, (b) RCCA, (c) SRCCA*_TT_*, (d) SRCCA*_WRST _*and (e) SRCCA*_WLT _*where the *X *and *Y *axes are the two most significant embedding components produced by the 3 different algorithms. CCA (a) suffers from lack of regularization, RCCA (b) is regularized but does not produce the best metaspace while the three variations of SRCCA (c)(d)(e) result in the best embedding components in terms of classification accuracy distinguished via best fit ellipses with one outlier.

These results, which seem to suggest that SRCCA outperforms the other two CCA based approaches for this dataset, CCA and RCCA, are observable in the embedding plots of Figure 2, which show the metaspace produced by CCA, RCCA, SRCCA*_TT_*, SRCCA*_WRST _*and SRCCA*_WLT _*with d = 2 components. It may be seen that because CCA lacks regularization, the corresponding covariance matrices are singular and lack inverses. For this reason, in Figure 2 the embedding components are not orthogonal but are highly correlated to each other and yield the same information. RCCA overcomes this regularization problem but still does not produce the same level of discrimination between patient classes compared to the 3 variations of SRCCA. Note that SRCCA*_TT_*, SRCCA*_WRST _*and SRCCA*_WLT _*chose similar regularization parameters, *λ_x _*and *λ_y_*, and have similar embedding plots.

### Experiment 2

We see that SRCCA*_TT_*, SRCCA*_WRST_*, SRCCA*_WLT _*are able to outperform PCA and PLSR in all 36 of 36 direct comparisons (see Tables 5 and 6). Even though, across both classifiers for d = 3, PCA and PLSR have median classification accuracies of 64% and 61%, which is higher than the accuracies for CCA and RCCA, it is still much lower than the 80% for SRCCA*_TT_*, SRCCA*_WRST_*, SRCCA*_WLT_*. These results also seem to indicate that SRCCA*_TT_*, SRCCA*_WRST_*, SRCCA*_WLT _*could also create a more appropriate metaspace than, not only CCA and RCCA, but also PCA and PLSR.

**Table 5:** Experiment 2: Classification Accuracy with K-Nearest Neighbor

Dataset (*ϕ*^*P *^, *ϕ*^*J *^)	PCA	PLSR	SRCCA*_TT_*	SRCCA*_WRST_*	SRCCA*_WLT_*
(*ϕ*^*P *^, *ϕ*^*M *^)	68%	57%	**80**%	79%	79%

(*ϕ*^*P *^, *ϕ*^*A *^)	63%	47%	**74**%	68%	**74**%

(*ϕ*^*P *^, *ϕ*^*H *^)	53%	53%	**74**%	**74**%	**74**%

Classification accuracies obtained for fusing (*ϕ*^*P *^, *ϕ*^*M *^), (*ϕ*^*P *^, *ϕ*^*A *^), and (*ϕ*^*P *^, *ϕ*^*H *^), with CCA, RCCA, SRCCA*_TT_*, SRCCA*_WRST_*, and SRCCA*_WLT _*using the top d = 3 components, using *ϕ*^*KNN *^with K = 1 neighbor and leave-one-out validation to identify patients at the risk of biochemical recurrence from those who are not.

**Table 6:** Experiment 2: Classification Accuracy with Random Forest

Dataset (*ϕ*^*P *^, *ϕ*^*J *^)	PCA	PLSR	SRCCA*_TT_*	SRCCA*_WRST_*	SRCCA*_WLT_*
(*ϕ*^*P *^, *ϕ*^*M *^)	64%	75%	83%	81%	**84**%

(*ϕ*^*P *^, *ϕ*^*A *^)	50%	64%	81%	77%	**83**%

(*ϕ*^*P *^, *ϕ*^*H *^)	64%	67%	91%	89%	**93**%

Classification accuracies obtained for fusing (*ϕ*^*P *^, *ϕ*^*M *^), (*ϕ*^*P *^, *ϕ*^*A *^), and (*ϕ*^*P *^, *ϕ*^*H *^), with CCA, RCCA, SRCCA*_TT_*, SRCCA*_WRST_*, and SRCCA*_WLT _*using the top d = 3 components, using *ϕ*^*RF *^with 50 trees and leave-one-out validation to identify patients at the risk of biochemical recurrence from those who are not.

### Experiment 3

In Tables 7 and 8 we see that the maximum and median *ϕ*^*KNN *^and *ϕ*^*RF *^of the 3 SRCCA variants for fusion of (*ϕ*^*I *^, *ϕ*^*J *^) were much higher than the corresponding values of PCA, PLSR, CCA or RCCA. We also see that SRCCA*_WLT _*attains a maximum classifier accuracy of 93.16% (see Table 7). In Tables 9 and 10, the 3 SRCCA variants are statistically significantly better than PCA, PLSR, CCA or RCCA even at the *p *= .001 level using either classifiers, *ϕ*^*KNN *^or *ϕ*^*RF *^. We further see that SRCCA*_WLT _*tends to marginally outperform SRCCA*_TT _*and SRCCA*_WRST_*. However given the small sample size it is difficult to draw any definitive conclusions about which of SRCCA*_TT_*, SRCCA*_WRST_*, or SRCCA*_WLT _*might be the better SRCCA variant.

**Table 7:** Experiment 3: Maximum *ϕ*^*KNN *^and *ϕ*^*RF *^of DF schemes across d ∈ {1, 2, ..10}

Classifier	PCA	PLS	CCA	RCCA	SRCCA*_TT_*	SRCCA*_WRST_*	SRCCA*_WLT_*
*ϕ*^*KNN *^	**84.21%**	**84.21%**	73.68%	68.42%	**84.21%**	**84.21%**	**84.21**%

*ϕ*^*RF *^	84.21%	84.21%	80.20%	68.42%	91.05%	88.95%	**93.16**%

Maximum classification accuracies obtained for fusing (*ϕ*^*P *^, *ϕ*^*M *^), (*ϕ*^*P *^, *ϕ*^*A *^), and (*ϕ*^*P *^, *ϕ*^*H *^), with PCA, PLSR, CCA, RCCA, SRCCA*_TT_*, SRCCA*_WRST_*, and SRCCA*_WLT _*across d ∈ {1, 2, ..10} components, using two classifiers, *ϕ*^*KNN *^, with K = 1, and *ϕ*^*RF *^, with 50 trees, and leave-one-out validation to identify patients at the risk of biochemical recurrence from those who are not.

**Table 8:** Experiment 3: Median *ϕ*^*KNN *^and *ϕ*^*RF *^of DF schemes across d ∈ {1, 2, ..10}

Classifier	PCA	PLS	CCA	RCCA	SRCCA*_TT_*	SRCCA*_WRST_*	SRCCA*_WLT_*
*ϕ*^*KNN *^	52.63%	57.89%	57.89%	47.37%	**68.42**%	**68.42**%	**68.42**%

*ϕ*^*RF *^	51.58%	62.37%	58.42%	37.37%	72.89%	69.47%	**74.21**%

Median classification accuracies obtained for fusing (*ϕ*^*P *^, *ϕ*^*M *^), (*ϕ*^*P *^, *ϕ*^*A *^), and (*ϕ*^*P *^, *ϕ*^*H *^), with PCA, PLSR, CCA, RCCA, SRCCA*_TT_*, SRCCA*_WRST_*, and SRCCA*_WLT _*across d ∈ {1, 2, ...10} components, using two classifiers, *ϕ*^*KNN *^, with K = 1, and *ϕ*^*RF *^, with 50 trees, and leave-one-out validation to identify patients at the risk of biochemical recurrence from those who are not.

**Table 9:** Experiment 3: Statistical Significance (p-value) of SRCCA for *ϕ*^*KNN *^

Classifier	SRCCA*_TT_*	SRCCA*_WRST_*	SRCCA*_WLT_*
PCA	5.9 × 10^-10^	9.0 × 10^-09^	4.7 × 10^-8^

PLS	6.0 × 10^-7^	9.2 × 10^-5^	2.2 × 10^-6^

CCA	3.0 × 10^-8^	1.3 × 10^-6^	4.0 × 10^-9^

RCCA	4.0 × 10^-10^	4.5 × 10^-10^	7.1 × 10^-11^

*p*-values for the twelve comparisons of every scheme in {PCA, PLSR, CCA, RCCA} to every scheme in {SRCCA*_TT_*, SRCCA*_WRST_*, SRCCA*_WLT_*} for fusing (*ϕ*^*P *^, *ϕ*^*M *^), (*ϕ*^*P *^, *ϕ*^*A *^), and (*ϕ*^*P *^, *ϕ*^*H *^) across d ∈ {1, 2, ...10} components, using two classifiers, *ϕ*^*KNN *^, with K = 1, and leave-one-out validation to identify patients at the risk of biochemical recurrence from those who are not.

**Table 10:** Experiment 3: Statistical Significance (p-value) of SRCCA for *ϕ*^*RF *^

Classifier	SRCCA*_TT_*	SRCCA*_WRST_*	SRCCA*_WLT_*
PCA	1.7 × 10^-13^	4.7 × 10^-12^	1.4 × 10^-10^

PLS	1.3 × 10^-5^	8.5 × 10^-3^	1.6 × 10^-4^

CCA	6.8 × 10^-7^	5.4 × 10^-6^	2.1 × 10^-7^

RCCA	3.4 × 10^-9^	1.8 × 10^-9^	3.6 × 10^-16^

*p*-values for the twelve comparisons of every scheme in {PCA, PLSR, CCA, RCCA} to every scheme in {SRCCA*_TT_*, SRCCA*_WRST_*, SRCCA*_WLT_*} for fusing (*ϕ*^*P *^, *ϕ*^*M *^), (*ϕ*^*P *^, *ϕ*^*A *^), and (*ϕ*^*P *^, *ϕ*^*H *^) across d ∈ {1, 2, ...10} components, using two classifiers, *ϕ*^*RF *^, with 50 trees, and leave-one-out validation to identify patients at the risk of biochemical recurrence from those who are not.

**Figure 3:**
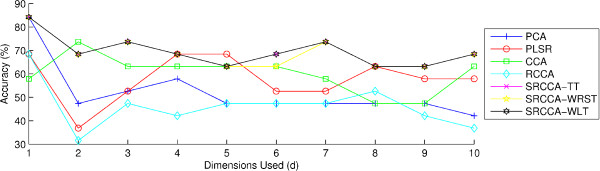
**Classification accuracies of (*ϕ*^*P *^, *ϕ*^*H *^) across dimensions d ∈ {1, 2, ..10} using the classifier *ϕ*^*KNN *^**. Accuracies were obtained for fusing (*ϕ*^*P *^, *ϕ*^*H *^), with PCA, PLSR, CCA, RCCA, SRCCA*_TT_*, SRCCA*_WRST_*, and SRCCA*_WLT _*across d ∈ {1, 2, ...10} components, using *ϕ*^*KNN *^, with K = 1, and leave-one-out validation to identify patients at the risk of biochemical recurrence from those who are not.

**Figure 4:**
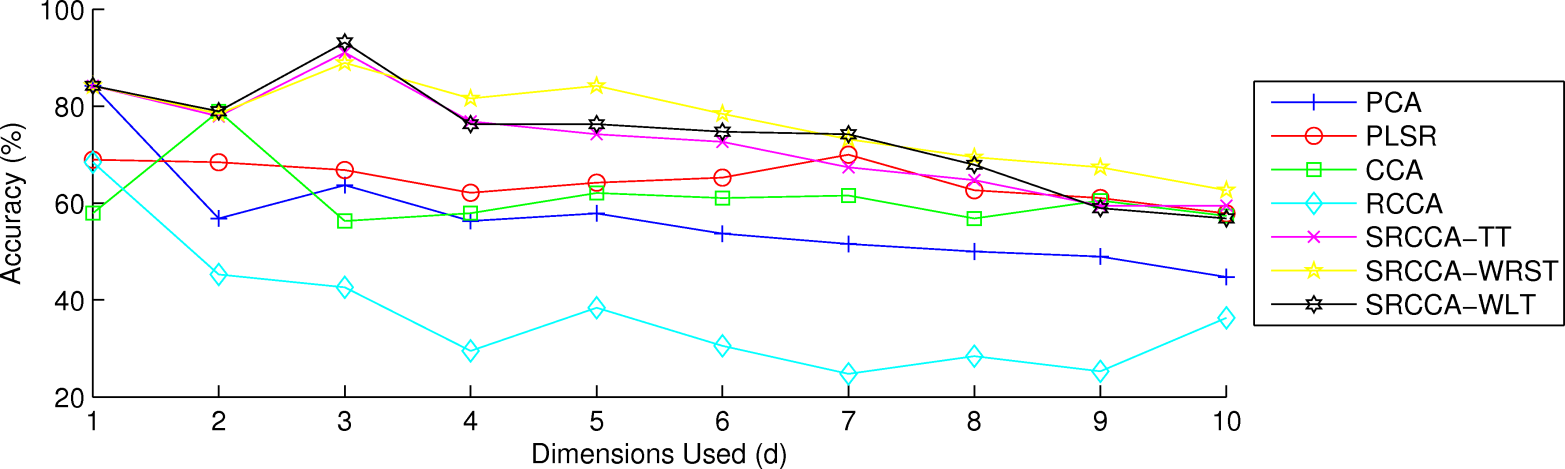
**Classification accuracies of (*ϕ*^*P *^, *ϕ*^*H *^) across dimensions d ∈ {1, 2, ..10} using the classifier *ϕ*^*RF *^**. Accuracies were obtained for fusing (*ϕ*^*P *^, *ϕ*^*H *^), with PCA, PLSR, CCA, RCCA, SRCCA*_TT_*, SRCCA*_WRST_*, and SRCCA*_WLT _*across d ∈ {1, 2, ...10} components, using *ϕ*^*KNN *^, with K = 1, and leave-one-out validation to identify patients at the risk of biochemical recurrence from those who are not.

In Figures 3 and 4, we see the classification accuracies of the 7 DF methods, PCA, PLSR, CCA, RCCA, SRCCA*_TT_*, SRCCA*_WRST_*, or SRCCA*_WLT _*over a range of d ∈ {1, 2, ..10} embedding components for the fusion (*ϕ*^*P *^, *ϕ*^*H *^). Importantly, we see that the SRCCA*_TT_*, SRCCA*_WRST_*, and SRCCA*_WLT _*all outperform PCA, PLSR, CCA and RCCA for a majority of the embedding dimensions, across both the *ϕ*^*KNN *^and *ϕ*^*RF *^classifiers.

### Experiment 4

**Figure 5:**
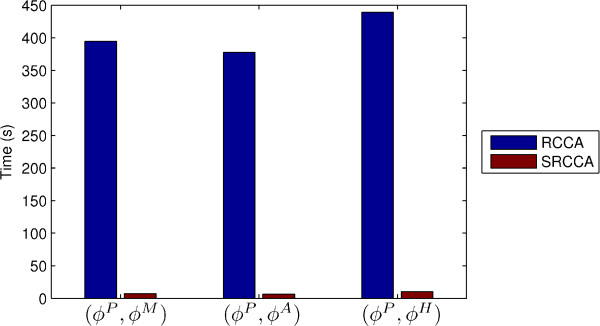
**Computational run times for SRCCA and RCCA for fusing (*ϕ*^*P *^, *ϕ*^*M *^), (*ϕ*^*P *^, *ϕ*^*A *^), and (*ϕ*^*P *^, *ϕ*^*H *^). SRCCA significantly outperforms RCCA across all fusion experiments**. SRCCA significantly outperforms RCCA across all fusion experiments.

Figure 5 reveals that the completion time of SRCCA is significantly lower than the completion time of RCCA. Even though the differences in these times are visibly different, a *p*-value of 1.9 × 10^-3 ^even with just 3 samples, indicates that SRCCA appears to be statistically significantly faster compared to RCCA.

Note that the canonical factorization stage is the most time consuming part of the of the algorithm. The Feature Selection stage computation, in comparison, is not as time consuming. SRCCA*_TT_*, SRCCA*_WRST_*, and SRCCA*_WLT _*(whose results are reported in Figure 5) all have similar execution times.

## Conclusions

In this paper, we presented a novel data fusion (DF) algorithm called Supervised Regularized Canonical Correlation Analysis (SRCCA) that, unlike CCA and RCCA, is (1) able to fuse with a feature selection (FS) scheme, (2) regularized, and (3) computationally cheap. We demonstrate how SRCCA can be used for quantitative integration and representation of multi-scale, multi-modal imaging and non-imaging data. In this work we leveraged SRCCA for the purpose of constructing a fused quantitative histologic-proteomic classifier for predicting which prostate cancer patients are at risk for 5 year biochemical recurrence following surgery. We have demonstrated that SRCCA is able to (1) produce a metaspace, where the samples are more stratified than the metaspace produced by CCA or RCCA, (2) better identify patients at the risk of biochemical recurrence compared to Principal Component Analysis (PCA), Partial Least Squares Regression (PLSR), CCA or RCCA, (3) perform regularization, all the while being statistically significantly faster compared to RCCA.

While the fused prognostic classifier for predicting biochemical recurrence in this work appears to be promising, we also acknowledge the limitations of this work: (1) As previously mentioned, the cost of mass spectrometry limited this study to only 19 datasets. By using a minimum sample size derivation model [70,71], we were able to determine that our fused SRCCA classifier would yield an accuracy of 93%, more than 95% of the time if our dataset were expanded to 56 studies. We intend to evaluate our classifier on such a cohort in the future. (2) Ideally, a randomized cross validation strategy should have been employed for the training and evaluation of the classifier. Unfortunately, this was also limited by the size of the cohort. While both parametric and non-parametric feature selection strategies were employed in this work, the availability of a larger dataset for classification in conjunction with SRCCA would allow for employment of parametric selection strategies, assuming that the underlying distribution can be estimated. For small sample datasets, a non-parametric feature selection strategy might be more approrpriate. In future work, we also plan to apply SRCCA in the context of data fusion for other imaging and non-imaging datasets in the context of other problem domains and applications.

## Abbreviations

DF: Data Fusion; CCA: Canonical Correlation Analysis; RCCA: Regularized Canonical Correlation Analysis; SRCCA: Supervised Regularized Canonical Correlation Analysis; PCA: Principal Component Analysis; PLSR: Partial Least Squares Regression; DR: Dimensional Reduction; CaP: Prostate Cancer; PSA: Prostate Specific Antigen; MS: Mass Spectrometry; *ϕ*^*KNN *^: K-Nearest Neighbor; *ϕ*^*RF*^: Random Forest.

## Authors' contributions

AM and AG devised the methodology and formulated the experiments. AG drafted the manuscript in collaboration with GL. AM edited the manuscript. SRM, MDF, JET, and JWS provided the data and the clinical expertise. All authors have read and approved the final manuscript.
